# Effects of Combining Online Anodal Transcranial Direct Current Stimulation and Gait Training in Stroke Patients: A Systematic Review and Meta-Analysis

**DOI:** 10.3389/fnhum.2021.782305

**Published:** 2021-12-10

**Authors:** Tsubasa Mitsutake, Takeshi Imura, Tomonari Hori, Maiko Sakamoto, Ryo Tanaka

**Affiliations:** ^1^Department of Physical Therapy, Fukuoka International University of Health and Welfare, Fukuoka, Japan; ^2^Department of Rehabilitation, Faculty of Health Sciences, Hiroshima Cosmopolitan University, Hiroshima, Japan; ^3^Department of Rehabilitation, Fukuyama Rehabilitation Hospital, Hiroshima, Japan; ^4^Education and Research Centre for Community Medicine, Faculty of Medicine, Saga University, Saga, Japan; ^5^Graduate School of Humanities and Social Sciences, Hiroshima University, Hiroshima, Japan

**Keywords:** transcranial direct current stimulation, gait training, combination, online stimulation, stroke

## Abstract

**Objective:** Combining transcranial direct current stimulation (tDCS) and repetitive gait training may be effective for gait performance recovery after stroke; however, the timing of stimulation to obtain the best outcomes remains unclear. We performed a systematic review and meta-analysis to establish evidence for changes in gait performance between online stimulation (tDCS and repetitive gait training simultaneously) and offline stimulation (gait training after tDCS).

**Methods:** We comprehensively searched the electronic databases Medline, Cochrane Central Register of Controlled Trials, Physiotherapy Evidence Database, and Cumulative Index to Nursing and Allied Health Literature, and included studies that combined cases of anodal tDCS with motor-related areas of the lower limbs and gait training. Nine studies fulfilled the inclusion criteria and were included in the systematic review, of which six were included in the meta-analysis.

**Result:** The pooled effect estimate showed that anodal tDCS significantly improved the 10-m walking test (*p* = 0.04; *I*^2^ = 0%) and 6-min walking test (*p* = 0.001; *I*^2^ = 0%) in online stimulation compared to sham tDCS.

**Conclusion:** Our findings suggested that simultaneous interventions may effectively improve walking ability. However, we cannot draw definitive conclusions because of the small sample size. More high-quality studies are needed on the effects of online stimulation, including various stimulation parameters.

## Introduction

Stroke often causes walking problems due to sensorimotor dysfunction, such as motor paresis, decreased muscle strength, and impaired proprioceptive capabilities. Patients with stroke may experience a decreased quality of life (QOL) and limited activities of daily living because of disease-related walking dysfunction. Walking speed is the most common measure of walking ability and is one of the predictors of independence, mortality, functional status at home and in the community, and QOL (Wonsetler and Bowden, 2017). Therefore, improving mobility, including the walking speed, is an important goal for patients with stroke.

Regarding gait rehabilitation methods, repetitive gait training has presented beneficial effects in improving mobility. Body weight-supported treadmill training (BWSTT) has the potential to facilitate symmetrical gait training and promote cortical activities after stroke (Oh et al., 2021). In addition, robot-assisted gait training (RAGT) is effective for improving neuroplastic and clinical outcomes in individuals with hemiparetic stroke (Kim et al., 2020). A systematic review of the current guidelines showed that RAGT is generally recommended to improve lower limb motor function, including gait and strength (Calabró et al., 2021). These repetitive walking exercises are expected to induce cortical motor plasticity and improve mobility.

Transcranial direct current stimulation (tDCS) is another intervention that has the potential to greatly assist stroke rehabilitation (Hordacre et al., 2018; Klamroth-Marganska, 2018). TDCS is a non-invasive brain stimulation (NIBS) method that may promote motor function in patients with stroke by modulating cortical excitability (Nitsche and Paulus, 2001; Liew et al., 2014). Previous studies have reported that applying anodal tDCS over the lower extremity area of the primary motor cortex significantly improves force steadiness (Montenegro et al., 2016) as well as motor cortex excitability and function (Chang et al., 2015). The safety and effectiveness of tDCS technology have been proven in the treatment of various conditions (Lefaucheur et al., 2017).

Given the independent effectiveness of tDCS and repetitive gait training, the combination of these methods may be more effective than using them separately for gait performance recovery. Previous studies on brain function have showed that brain activity during walking increased bilaterally in the medial primary sensory and supplementary motor cortices (Miyai et al., 2001). Moreover, anodal stimulation of the primary motor cortex with tDCS increased cortical excitability (Nitsche and Paulus, 2001). Thus, gait training with simultaneous tDCS may improve gait performance *via* the re-enforced learning of neural networks, including the primary motor cortex.

Previous systematic reviews have showed that NIBS combined with other treatments improved various symptoms (Volz et al., 2012; Salazar et al., 2018; Cardenas-Rojas et al., 2020). Among them, the combination of tDCS and other therapies significantly improved gait performance in patients post-stroke (Vaz et al., 2019; Navarro-López et al., 2021). However, another review reported that there were no conclusive results supporting the role of tDCS in enhancing the effect of gait rehabilitation among patients with neurological disorders (de Paz et al., 2019). Improving balance performance by tDCS may limit the effects of tDCS on walking speed and/or walking endurance (Tien et al., 2020). The main limitation of previous tDCS reviews is the lack of uniformity in parameters, application patterns, and evaluation variables (de Paz et al., 2019; Santos et al., 2020). A potentially important aspect of the intervention method of combining tDCS with gait training is whether the treatments are applied simultaneously or after the stimulation. Previous studies have demonstrated an increase in corticospinal tract excitability by adapting robotic training after tDCS (Giacobbe et al., 2013; Powell et al., 2016). In contrast, another study showed that tDCS to bilateral primary motor areas while performing RAGT was more effective for the recovery of lower limb function in patients with stroke (Naro et al., 2021). Thus, it remains unclear which application timing of tDCS is optimal. Such detailed analysis of stimulus timing is novel in tDCS studies and may contribute to the establishment of effective intervention methods.

The objectives of this systematic review were to investigate the effects of the combination of anodal tDCS on motor-related areas and repetitive gait training, including BWSTT and RAGT on walking ability, and examine the differences between online stimulation, in which tDCS and repetitive gait training are performed simultaneously, and offline stimulation, in which tDCS is followed by gait training.

## Materials and Methods

A systematic review of the literature was performed according to the Preferred Reporting Items for Systematic reviews and Meta-Analysis Protocol (PRISMA) guidelines (Page et al., 2021). This review was registered with PROSPERO (ID: CRD42021247018).

### Eligibility Criteria

Studies were included in this systematic review if they met the following criteria: (1) the patients were diagnosed with hemorrhagic or ischemic stroke with unilateral hemiplegia; (2) the patients could walk without support and maintain their own body weight or balance; (3) a combination of anodal tDCS on the motor-related areas and repetitive gait training was performed; (4) gait performance outcomes were assessed; (5) the study was a randomized controlled trial (RCT), crossover RCT, or high-quality comparative studies; (6) the study was a clinical trial with at least seven sessions per week; and (7) the article was written in English.

Studies that met the following criteria were excluded: (1) the study included patients with subarachnoid hemorrhages; (2) the study included patients with a higher brain dysfunction, such as unilateral spatial neglect, that may affect gait performance; (3) the study was a meta-analysis, review, or case study; or (4) the study had insufficient data to calculate the effect size for quantitative analysis.

### Information Sources

The electronic databases Medline, Cochrane Central Register of Controlled Trials, Physiotherapy Evidence Database (PEDro), and Cumulative Index to Nursing and Allied Health Literature were comprehensively searched. The searches were performed on March 19, 2021.

### Search Strategy

The search terms of “patient,” “intervention,” and “outcome” were combined with the “AND” operator. “Patient” was defined as patients with stroke. “Intervention” was defined as a combination of tDCS and gait training. “Outcome” was defined as gait performance. For each concept, we combined synonyms and Medical Subject Headings terms with the “OR” operator. There were no limits on the dates. An example of the search strategy used in the Medline database is provided in Supplementary Table 1.

### Study Selection

The articles identified through database searching were summarized into spreadsheets that were created using Microsoft Excel 2019. After duplicates were removed, two authors (TM and TI) independently screened each article based on the titles and abstracts using predetermined eligibility criteria in order to determine relevant manuscripts for full-text review. Subsequently, full-text copies of articles that were not excluded based on the titles or abstracts were retrieved, and the inclusion and exclusion criteria were reapplied to these studies to determine their suitability for the final inclusion. Any disagreements at the article screening and selection stages were resolved through discussion, and decisions were made by a third party (RT) to reach a consensus.

### Data Collection Process

We prepared and used simple predesigned spreadsheets that were created using Microsoft Excel 2019 to extract data on participants, interventions, outcome measurements, and results. Two authors (TM and TI) discussed and decided whether the outcomes reported in the extracted studies corresponded to kinetic or kinematic measurements.

### Data Items

The following outcome measures were chosen for our meta-analysis: (1) 10-m walking test (10 MWT), which examines the walking speed; (2) 6-min walking test (6 MWT), which examines the walking endurance; (3) Functional Ambulatory Category (FAC), which examines the walking independence and functional ambulation; (4) walking cadence (the number of steps per minute), which examines the quality of walking ability; and (5) timed up and go test (TUGT), which examines functional mobility.

### Risk of Bias Evaluation in Individual Studies

To evaluate the risk of bias in each trial (de Morton, 2009), two researchers (TM and HT) independently applied the PEDro scale (Verhagen et al., 1998). Any differences in items were resolved through a discussion and decided by the agreement of a third party (TI). Studies were considered to be of high and moderate quality when the PEDro score was ≥6 and 4 or 5, respectively (Maher et al., 2003; Wallis and Taylor, 2011). Studies with scores <4 were excluded from further analysis (Van Peppen et al., 2004; Veerbeek et al., 2014; Kwakkel et al., 2015).

### Effect Measures

Regarding continuous outcomes, if the unit of measurements was consistent across trials, the results were presented as the weighted mean difference (MD) with 95% confidence intervals (95% CIs). If the outcome did not use the same units across studies, we used the standardized mean difference (SMD) instead of the MD.

### Synthesis Methods

All statistical comparisons were performed using Review Manager, version 5.4 (Cochrane Collaboration, London, United Kingdom). The included studies were selected to perform anodal tDCS to the motor-related areas of the lower limbs, as well as repetitive gait training and sham stimulation in the intervention and control groups, respectively. In addition, we included intervention studies with two or more of the same assessment methods. This meta-analysis used the mean and standard deviation of the difference in the values obtained pre- and post-intervention. When the means and standard deviations were not provided in the manuscript, we used the post-intervention values. Moreover, the 10 MWT evaluated in time (s) was transformed into a negative value to be consistent with the values evaluated in speed (m/s). This meta-analysis excluded those studies that measured walking speed by means other than the 10 MWT to quantitatively assess walking ability.

We analyzed the effect of anodal tDCS on gait performance, followed by a subgroup analysis with online or offline stimuli. Subgroup meta-analysis was possible when at least two studies with a similar design were available for each stimulus group. A random-effects model was used to account for differences in effect sizes between the studies (Borenstein et al., 2010). Statistical heterogeneity was assessed using the *I*^2^ statistic. *I*^2^ values >25 and 50% were considered indicative of moderate and high heterogeneity, respectively. Statistical significance was set at *p* < 0.05.

### Certainty Assessment

The quality of evidence for each evaluation parameter was assessed using the Grading of Recommendations Assessment, Development and Evaluation (GRADE) system (Guyatt et al., 2011). The GRADE system was implemented when there were at least two applicable outcomes. The quality of evidence was assessed as “very low,” “low,” “moderate,” or “high” based on certain criteria. Factors downgrading the quality (risk of bias, inconsistency, indirectness, impression, and publication bias) or upgrading the quality (large effect, plausible confounding, and dose-response) were evaluated (Yamakawa et al., 2015).

## Results

### Search Selection

**FIGURE 1:**
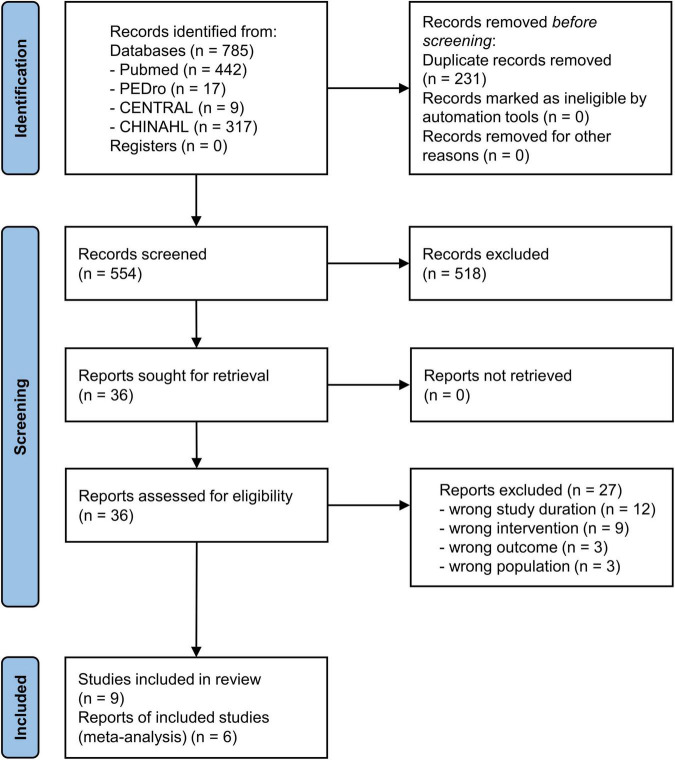
Flow diagram of the study selection process.

The combined database search identified 785 trials (Figure 1). After adjusting for duplicates, 554 trials were included in the analyses, of which 518 did not meet the selection criteria on reviewing the article titles and abstracts. The complete text of the remaining 36 studies were examined in detail. Twenty-seven studies did not meet the inclusion criteria. Finally, nine studies fulfilled the inclusion criteria and were included in the systematic review (Geroin et al., 2011; Picelli et al., 2015, 2018; Leon et al., 2017; Seo et al., 2017; Manji et al., 2018; Madhavan et al., 2020; Mitsutake et al., 2021; Naro et al., 2021). Then, six of these studies were included in the meta-analysis (Geroin et al., 2011; Picelli et al., 2015; Seo et al., 2017; Manji et al., 2018; Madhavan et al., 2020; Mitsutake et al., 2021). The critical information of the six studies are summarized in Table 1, including the study population, tDCS parameters, intervention methods, and main outcomes.

**TABLE 1:** Summary of included studies.

Study (Author, Journal, Year)	Study design	Size N (IG/CG)	Age Mean ± SD (IG/CG)	Sex M/F	Time since stroke	Intervention	Area of stimulation	Current density	Sessions; intervals	Stimulation timing	Order of application	Outcome measures
Naro et al., 2021	Retrospective	IG: 9 OG1: 15 OG2: 13	IG: 68 ± 4 OG1: 66 ± 5 OG2: 72 ± 4	IG: 4/5 OG1: 6/9 OG2: 5/8	IG: 10 ± 2 m OG1: 11 ± 3 m OG2: 8 ± 2 m	IG: dstDCS + RAGT (on-RAGT) OG1: dstDCS + RAGT (post-RAGT) OG2: dstDCS + RAGT (pre-RAGT)	A; PMA-affected side (C3/4) C; PMA-non affected side (C3/4)	2.0 mA 35 cm^2^ 0.057 mA/cm^2^	48 s; 8 weeks	ONLINE	dstDCS (first 10 min) + RAGT (50 min)	10 MWT; 6 MWT; FAC; MI; Tinetti scale; FIM; MEP
Picelli et al., 2018	RCT	IG: 10 OG: 10	IG: 62.6 ± 8.3 OG: 62.8 ± 11.8	IG: 7/3 OG: 6/4	IG: 67.1 ± 46.8 m OG: 51.9 ± 41.2 m	IG: Anodal tDCS + tsDCS + RAGT OG: Cathodal tcDCS + tsDCS + RAGT	IG: A; PMA-affected LE (Cz), C; OA-CL CG: A; cerebellar hemisphere (O1/2), C; buccinator muscle-IL	2.0 mA 12.56 cm^2^ 0.159 mA/cm^2^	10 s; 2 weeks	ONLINE	tDCS (20 min) + RAGT (20 min)	6 MWT; FAC; MI; Gait analysis (cadence); ashworth scale
Picelli et al., 2015	RCT	IG: 10 CG: 10 OG: 10	IG: 62.8 ± 11.8 CG: 61.0 ± 7.2 OG: 64.8 ± 6.0	IG: 7/3 CG: 8/2 OG: 7/3	IG: 51.9 ± 41.1 m CG: 54.8 ± 32.9 m OG: 61.3 ± 29.3 m	IG: Anodal tDCS + tsDCS + RAGT CG: Sham tDCS + tsDCS + RAGT OG: Anodal tDCS + sham tsDCS + RAGT	A; PMA-affected side (C3/4) C; OA-CL	2.0 mA 35 cm^2^ 0.057 mA/cm^2^	10 s; 2 weeks	ONLINE	tDCS (20m in) + RAGT (20 min)	6 MWT; FAC; MI; Gait analysis (cadence); ashworth scale
Geroin et al., 2011	RCT	IG: 10 CG: 10 OG: 10	IG: 63.6 ± 6.7 CG: 63.3 ± 6.4 OG: 61.1 ± 6.3	IG: 8/2 CG: 6/4 OG: 9/1	IG: 25.7 ± 6.0 m CG: 26.7 ± 5.1 m OG: 26.9 ± 5.8 m	IG: Anodal tDCS + RAGT CG: Sham tDCS + RAGT OG: Walking exercises	A; PMA-affected LE C; SOA-CL	1.5 mA 35 cm^2^ 0.043 mA/cm^2^	10 s; 2 weeks	ONLINE	tDCS (7 min) + RAGT (20 min)	10 MWT; 6MWT; FAC; MI; Gait analysis (cadence); RMI
Madhavan et al., 2020	RCT	IG: 21 CG: 20 OG1: 20 OG2: 20	IG: 58 ± 11 CG: 58 ± 10 OG1: 60 ± 9 OG2: 59 ± 9	IG: 14/7 CG: 11/9 OG1: 15/5 OG2: 15/5	IG: 4.3 ± 3.6 y CG: 6.1 ± 4.2 y OG1: 5.6 ± 3.6 y OG2: 5.9 ± 5.6 y	IG: Anodal tDCS + HISTT CG: Sham tDCS + HISTT OG1: AMT + HISTT OG2: tDCS + AMT + HISTT	A; PMA-affected LE C; SOA-CL	1.0 mA 12.5 cm^2^ 0.080 mA/cm^2^	12 s; 4 weeks	OFFLINE	tDCS (15 min) → HISTT (40 min)	10 MWT; 6 MWT; BBS; TUGT; mini-BESTest; ABC; FMA; SIS; MEP
Manji et al., 2018	Crossover	IG: 15 CG: 15	IG: 62.2 ± 10.1 CG: 63.7 ± 11.0	IG: 10/5 CG: 11/4	IG: 134.5 ± 55.7 d CG: 149.7 ± 24.2 d	IG: Anodal tDCS + BWSTT CG: Sham tDCS + BWSTT	A; SMA (3.5 cm anterior to Cz) C; EOC	1.0 mA 25 cm^2^ 0.040 mA/cm^2^	7 s; 1 week	ONLINE	tDCS (20 min) + BWSTT (20 min)	10 MWT; TUGT; FMA; POMA; TCT
Seo et al., 2017	RCT	IG: 11 CG: 10	IG: 61.1 ± 8.9 CG: 62.9 ± 8.9	IG: 9/2 CG: 7/3	IG: 75.5 ± 83.4 m CG: 152.5 ± 122.8 m	IG: Anodal tDCS + RAGT CG: Sham tDCS + RAGT	A; PMA-affected LE C; SOA-CL	2.0 mA 35 cm^2^ 0.057 mA/cm^2^	10 s; 2 weeks	OFFLINE	tDCS (20 min) → RAGT (45 min)	10 MWT; 6 MWT; FAC; BBS; FMA; MRCS; MEP
Leon et al., 2017	Active control	IG: 10 CG: 23 OG: 17	IG: 49 ± 9 CG: 49 ± 11 OG: 47 ± 11	IG: 6/4 CG: 17/6 OG: 12/5	IG: 53 ± 25 d CG: 64 ± 33 d OG: 56 ± 38 d	IG: Anodal tDCS + RAGT CG: Sham tDCS + RAGT OG: Anodal tDCS (C3/4) + RAGT	A; Cz C; SOA-Rt	2.0 mA 35 cm^2^ 0.057 mA/cm^2^	20 s; 4 weeks	ONLINE	tDCS (first 20 min) + RAGT (30 – 45 min)	10 MWT; FAC
Mitsutake et al., 2021	RCT	IG: 11 CG: 12 OG: 11	IG: 74.9 ± 9.2 CG: 67.3 ± 12.1 OG: 75.6 ± 11.0	IG: 6/5 CG: 9/3 OG: 4/7	IG: 44.6 ± 31.7 d CG: 37.1 ± 27.3 d OG: 34.6 ± 17.8 d	IG: Anodal tDCS + FES CG: Sham tDCS + FES OG: Anodal tDCS	A; PMA-affected LE C; SOA-CL	2.0 mA 35 cm^2^ 0.057 mA/cm^2^	7 s; 1 week	ONLINE	tDCS (20 min) + FES (20 min)	10 MWT; Gait analysis (acceleration parameters)

*RCT, randomized controlled trial; IG, intervention group; CG, control group; OG, other group; tDCS, transcranial direct current stimulation; dstDCS, dual-site transcranial direct current stimulation; tcDCS, transcranial cerebellar direct current stimulation; tsDCS transcutaneous spinal direct current stimulation; RAGT, robot-assisted gait training; HISTT, high-intensity speed-based treadmill training; AMT, ankle motor tracking; BWSTT, body weight-supported treadmill training; FES, functional electrical stimulation; A, anode; C, cathode; PMA, primary motor area; LE, lower extremity; OA, orbital area; CL, contralateral side; IL, ipsilateral side; SOA, supra-orbital area; SMA, supplementary motor area; EOC, exterior occipital crest; 10 MWT, 10-m walking test; 6 MWT, 6-min walking test; FAC, functional ambulatory category; MI, mobility index; FIM, functional independence measure; MEP, motor evoked potential; RMI, rivermead mobility index; BBS, berg balance scale; TUG, timed up and go test; mini-BESTest, mini-balance evaluation systems test; FMA, fugl-meyer assessment; ABC, activities-specific balance confidence scale; SIS, stroke impact scale; POMA, performance-oriented mobility assessment; TIS, trunk controltest; MRCS, medical research council scale.*

### Study Characteristics

Six RCTs (Geroin et al., 2011; Picelli et al., 2015, 2018; Seo et al., 2017; Madhavan et al., 2020; Mitsutake et al., 2021), one crossover trial (Manji et al., 2018), one active-control article (Leon et al., 2017), and one retrospective clinical trial (Naro et al., 2021) were included in this study. The sample sizes ranged from 20 (Picelli et al., 2015, 2018) to 41 (Madhavan et al., 2020), and the participants were divided into the intervention and control groups. The average age of the participants of nine studies ranged from 49 (Leon et al., 2017) to 74.9 years (Mitsutake et al., 2021). In addition, the stroke phase at baseline ranged from 37.1 days (Mitsutake et al., 2021) to 152.5 months (Seo et al., 2017) after onset. Seven studies that reported results from the 10 MWT (Geroin et al., 2011; Leon et al., 2017; Seo et al., 2017; Manji et al., 2018; Madhavan et al., 2020; Mitsutake et al., 2021; Naro et al., 2021), six studies reported results from the 6 MWT (Geroin et al., 2011; Picelli et al., 2015, 2018; Seo et al., 2017; Madhavan et al., 2020; Naro et al., 2021), six studies reported FAC (Geroin et al., 2011; Picelli et al., 2015, 2018; Leon et al., 2017; Seo et al., 2017; Naro et al., 2021), three studies reported walking cadence (Geroin et al., 2011; Picelli et al., 2015, 2018), and two studies reported results from the TUGT (Manji et al., 2018; Madhavan et al., 2020).

The tDCS intensity was tested at 1.0, 1.5, or 2.0 mA (two, one, and six studies, respectively). The electrode size was 12.5, 25, or 35 cm^2^ (two, one, and six studies, respectively). Regarding the electrode placement locations, seven studies applied the anodal and cathodal electrodes in the motor-related area, including the primary motor area, and the orbital area, respectively (Geroin et al., 2011; Picelli et al., 2015, 2018; Leon et al., 2017; Seo et al., 2017; Madhavan et al., 2020; Mitsutake et al., 2021), while one study applied the anodal electrodes in the supplementary motor area and the cathodal electrodes in the exterior occipital crest (Manji et al., 2018). Another study performed dual-site tDCS with electrodes placed in the bilateral primary motor areas (Naro et al., 2021). Regarding the tDCS intervention methods in the control group, seven studies tested the combined effects of sham stimulation and repetitive walking training (Geroin et al., 2011; Picelli et al., 2015; Leon et al., 2017; Seo et al., 2017; Manji et al., 2018; Madhavan et al., 2020; Mitsutake et al., 2021), and one study compared anodal tDCS performed in the primary motor area with cathodal tDCS performed in the cerebellar hemispheres (tcDCS; Picelli et al., 2018). Moreover, one study compared the tDCS while performing walking training to that followed by walking training. The results showed that simultaneous intervention significantly improved walking endurance compared to gait training after tDCS (Picelli et al., 2018). Regarding the non-cortical tDCS intervention, two studies performed the tDCS combined with cathodal transcutaneous spinal direct current stimulation (tsDCS; Picelli et al., 2015, 2018). The combination of cathodal tcDCS and cathodal tsDCS during RAGT significantly improved walking endurance compared to the combination of anodal tDCS and cathodal tsDCS during RAGT (Picelli et al., 2018).

Concerning the repetitive walking training methods, six, one, one, and one studies performed RAGT (Geroin et al., 2011; Picelli et al., 2015, 2018; Leon et al., 2017; Seo et al., 2017; Naro et al., 2021), BWSTT (Manji et al., 2018), high-intensity speed-based treadmill training (HISTT; Madhavan et al., 2020), and functional electrical stimulation (FES; Mitsutake et al., 2021), respectively.

### Risk of Bias in Studies

The application of the PEDro scale revealed that all studies (Geroin et al., 2011; Picelli et al., 2015, 2018; Leon et al., 2017; Seo et al., 2017; Manji et al., 2018; Madhavan et al., 2020; Mitsutake et al., 2021; Naro et al., 2021) had good methodological quality and met the evaluation criteria with scores ≥6 deemed to contain good scientific evidence (Table 2).

**TABLE 2:** Methodological quality of included studies in accordance with the PEDro scores.

Study	1*	2	3	4	5	6	7	8	9	10	11	Total
Naro et al., 2021	✓			✓			✓	✓	✓	✓	✓	6/10
Picelli et al., 2018	✓	✓	✓	✓	✓		✓	✓	✓	✓	✓	9/10
Picelli et al., 2015	✓	✓	✓	✓	✓		✓	✓	✓	✓	✓	9/10
Geroin et al., 2011	✓	✓		✓	✓		✓	✓	✓	✓	✓	8/10
Madhavan et al., 2020	✓	✓	✓	✓	✓		✓	✓	✓	✓	✓	9/10
Manji et al., 2018	✓	✓		✓	✓	✓		✓	✓	✓	✓	8/10
Seo et al., 2017	✓	✓	✓	✓	✓	✓	✓		✓	✓	✓	9/10
Leon et al., 2017	✓			✓	✓			✓	✓	✓	✓	6/10
Mitsutake et al., 2021	✓	✓	✓	✓	✓		✓	✓		✓	✓	8/10

*Not included in the total score. PEDro scores: 1, eligibility criteria specified; 2, participants randomly allocated to groups; 3, allocation concealed; 4, groups similar at baseline; 5, participants were blinded; 6, therapists were blinded; 7, assessors were blinded; 8, data available for more than 85% of participants; 9, participants received the treatment as allocated or intention-to-treat analysis was used; 10, statistical analyses were reported; 11, point measures and variability measures of data reported.

### Results of Individual Studies and Syntheses

Six studies were divided broadly into two research categories: (1) online stimulation, in which anodal tDCS and repetitive gait training were performed simultaneously; and (2) offline stimulation, in which anodal tDCS was followed by gait training. Four and two studies used online (Geroin et al., 2011; Picelli et al., 2015; Manji et al., 2018; Mitsutake et al., 2021) and offline stimulation (Seo et al., 2017; Madhavan et al., 2020), respectively.

**FIGURE 2:**
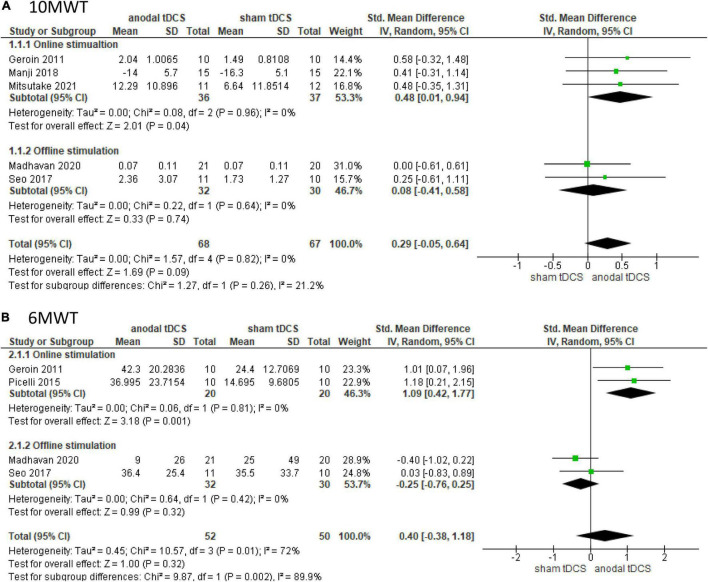
Forrest plot displaying the standardized mean differences (SMD) between anodal and sham tDCSs. **(A)** Subgroup analysis based on stimulus timing for the 10-m walking test (10 MWT). **(B)** Subgroup analysis based on stimulus timing for the 6-min walking test (6 MWT).

Five studies involving 135 patients were included in the meta-analysis of 10 MWT, of which three and two involved online and offline stimulation, respectively. The test for subgroup differences showed no statistically significant subgroup effect (*p* = 0.26), suggesting that stimulation timing did not modify the effect of anodal tDCS in comparison to sham tDCS. The pooled effect estimate favored anodal tDCS in online stimulation: anodal tDCS (*n* = 36) significantly increased the walking speed compared to sham tDCS (*n* = 37) (SMD: 0.48; 95% CI: 0.01–0.94; *p* = 0.04; *I*^2^ = 0%, Figure 2A). In the offline stimulation, the effect of anodal tDCS (*n* = 32) was not significantly different from that of sham tDCS (*n* = 30) (SMD: 0.08; 95% CI: −0.41, 0.58; *p* = 0.74; *I*^2^ = 0%, Figure 2A). After combining data from both online and offline stimulations, anodal tDCS (*n* = 68) did not significantly increase the walking speed compared to sham tDCS (*n* = 67) (SMD: 0.29; 95% CI: −0.05, 0.64; *p* = 0.09; *I*^2^ = 0%).

Four studies involving 102 patients were included in the meta-analysis of the 6 MWT, of which two tested online stimulation and two tested offline stimulation. The test for subgroup differences showed a statistically significant subgroup effect (*p* = 0.002). The pooled effect estimate showed that anodal tDCS (*n* = 20) significantly increased walking distance in online stimulation compared to sham tDCS (*n* = 20) (SMD: 1.09; 95% CI: 0.42–1.77; *p* = 0.001; *I*^2^ = 0%, Figure 2B). In the offline stimulation, the effect of anodal tDCS (*n* = 32) was not significantly different from that of sham tDCS (*n* = 30) (SMD: −0.25; 95% CI: −0.76–0.25; *p* = 0.32; *I*^2^ = 0%, Figure 2B). On combining data from both online and offline stimulations, anodal tDCS (*n* = 52) did not significantly increase walking distance compared to the sham tDCS (*n* = 50), and the studies presented high heterogeneity (SMD: 0.40; 95% CI: −0.38–1.18; *p* = 0.32; *I*^2^ = 72%).

**FIGURE 3:**
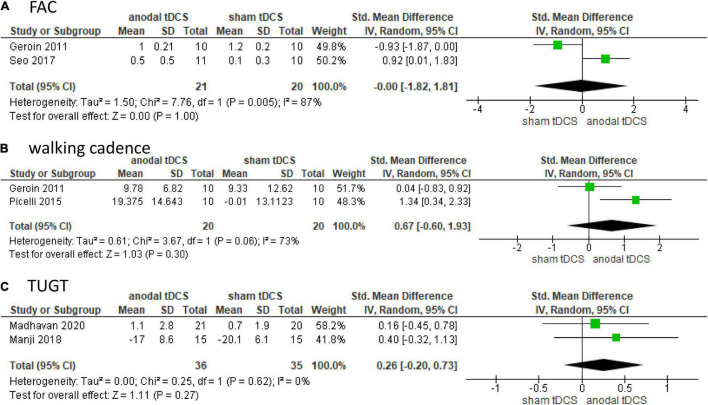
Forrest plot displaying the standardized mean differences (SMD) between anodal and sham tDCSs. **(A)** Functional Ambulatory Category (FAC). **(B)** Walking cadence. **(C)** Timed up and go test (TUGT).

Subgroup analysis was not performed for FAC, walking cadence, or TUGT because of the small number of included studies. Two studies involving 41 patients were included in the meta-analysis and showed no significant difference in FAC in the anodal tDCS compared to the sham tDCS (SMD: 0.00; 95% CI: −1.82–1.81; *p* = 1.00; *I*^2^ = 87%, Figure 3A).

Two studies involving 40 patients were included in the meta-analysis and showed no significant difference in walking cadence in the anodal tDCS compared to the sham tDCS (SMD: 0.67; 95% CI: −0.60–1.93; *p* = 0.30; *I*^2^ = 73%, Figure 3B).

Two studies involving 71 patients were included in the meta-analysis and showed no significant difference in TUGT in anodal tDCS compared to that in sham tDCS (SMD: 0.26; 95% CI: −0.20–0.73; *p* = 0.27; *I*^2^ = 0%, Figure 3C).

### Certainty of Evidence

When the quality of evidence was evaluated using the GRADE system, all parameters were rated from very low to moderate, with a risk of bias, inconsistency, and impression as factors that reduced quality. The 6 MWT was rated as “very low” for the combined online and offline data, but was rated as “moderate” for each item with inconsistencies indicated as “not serious” (Table 3).

**TABLE 3:** Summary of GRADE findings.

Certainty assessment									No. of patients		Certainty	Importance
Outcomes	Stimulation timing	No. of studies	Study design	Risk of bias	Inconsistency	Indirectness	Imprecision	Other considerations	Intervention group	Control group		
10 MWT	Online	3	RCT	Serious^a^	Not serious	Not serious	Not serious	None	36	37	⊕⊕⊕◯ Moderate	Important
	Offline	2	RCT	Not serious	Not serious	Not serious	Serious^f^	None	32	30	⊕⊕⊕◯ Moderate	Important
	Total	5	RCT	Serious^a^	Not serious	Not serious	Serious^f^	None	68	67	⊕⊕◯◯ Low	Important
6 MWT	Online	2	RCT	Serious^b^	Not serious	Not serious	Not serious	None	20	20	⊕⊕⊕◯ Moderate	Important
	Offline	2	RCT	Not serious	Not serious	Not serious	Serious^f^	None	32	30	⊕⊕⊕◯ Moderate	Important
	Total	4	RCT	Serious^b^	Serious^c^	Not serious	Serious^f^	None	52	50	⊕◯◯◯ Very low	Important
FAC	Online	1	RCT	Serious^b^	N.A.	Not serious	Not serious	None	10	10	N.A.	Important
	Offline	1	RCT	Not serious	N.A.	Not serious	Not serisous	None	11	10	N.A.	Important
	Total	2	RCT	Serious^b^	Serious^d^	Not serious	Serious^f^	None	21	20	⊕◯◯◯ Very low	Important
Gait analysis (cadence)	Online	2	RCT	Serious^b^	Serious^e^	Not serious	Serious^f^	None	20	20	⊕◯◯◯ Very low	Important
	Offline	–	–	–	–	–	–	–	–	–	–	–
	Total	2	RCT	Serious^b^	Serious^e^	Not serious	Serious^f^	None	20	20	⊕◯◯◯ Very low	Important
TUGT	Online	1	RCT	Serious^b^	N.A.	Not serious	Serious^f^	None	15	15	N.A.	Important
	Offline	1	RCT	Not serious	N.A.	Not serious	Serious^f^	None	21	20	N.A.	Important
	Total	2	RCT	Serious^b^	Not serious	Not serious	Serious^f^	None	36	35	⊕⊕◯◯ Low	Important

*N.A.: Not applicable, 10 MWT: 10-m walking test, 6 MWT: 6-min walking test, FAC: Functional ambulation category, TUGT: Timed up and go test, RCT: Randomized controlled trial.*

*^a^Indicating three studies with moderate risk of bias, ^b^Indicating one study with moderate risk of bias, ^c^I^2^ = 72%, ^d^I^2^ = 87%, ^e^I^2^ = 73%, and ^f^Wide 95% confidence interval.*

## Discussion

This systematic review aimed to investigate the effects of the combination of anodal tDCS on motor-related areas and repetitive gait training, including BWSTT and RAGT, on walking ability. Moreover, it aimed to examine the differences between online stimulation, in which tDCS and repetitive gait training are performed simultaneously, and offline stimulation, in which tDCS is followed by gait training.

Of the six studies that met the inclusion criteria for meta-analysis, four and two were classified as online and offline stimulation studies, respectively. The quality of the evidence, including the risk of bias described in these studies, was generally high, as assessed by the PEDro scale and GRADE criteria.

The results of the subgroup analysis showed that online stimulation significantly increased the distance in the 6 MWT compared to offline stimulation. Moreover, anodal tDCS significantly improved the results of the 10 MWT and 6 MWT compared to sham tDCS. The 10 MWT and 6 MWT are general indices of walking ability. Decreased cardiovascular fitness in stroke survivors may negatively impact their social life and QOL (Mayo et al., 1999). The walking speed of patients post-stroke is significantly related to walking performance, QOL, social participation, and even the ability to return to work (Suttiwong et al., 2018; Grau-Pellicer et al., 2019; Jarvis et al., 2019). The current meta-analysis showed that online stimulation may have greater effects on walking performance than offline stimulation. Interestingly, Naro et al. (2021) investigated the efficacy and safety of dual-site tDCS in the bilateral primary motor area timed with RAGT in patients with stroke. They showed that simultaneous intervention of tDCS and RAGT significantly improved gait endurance compared to RAGT after tDCS (Naro et al., 2021). Given that the combination of tDCS and repetitive gait training facilitates neuroplasticity in patients post-stroke through aerobic effort (Mellow et al., 2020), walking training with simultaneous tDCS may improve gait performance *via* the re-enforced learning of neural networks, including the primary motor area.

The test for the overall effect showed no significant difference in all parameters, including the 10 MWT, 6 MWT, FAC, walking cadence, or TUGT. Madhavan et al. (Madhavan et al., 2020) reported that combining HISTT and tDCS to the primary motor cortex at a current intensity of 1.0 mA did not improve walking speed or secondary behavioral outcome measures. Geroin et al. (2011) also showed that tDCS to the primary motor cortex at a current intensity of 1.5 mA had no additional effect on RAGT. Interestingly, these studies (Geroin et al., 2011; Madhavan et al., 2020) tended to have shorter tDCS intervention times compared to those of the other studies (Picelli et al., 2015, 2018; Leon et al., 2017; Seo et al., 2017; Manji et al., 2018; Mitsutake et al., 2021). The motor cortex of the lower limb is located deep between both hemispheres and requires higher intensity stimulation compared to the upper limb (Jeffery et al., 2007). Applying anodal tDCS to the ipsilateral hemisphere may not help recovery in all individuals, and neuromodulation interventions should be individually tailored (Liew et al., 2014; Plow et al., 2016). These findings suggested that tDCS may decrease cortical excitability and limit the effects of priming (Wiethoff et al., 2014; Madhavan et al., 2016), indicating that the tDCS intervention should be carefully observed for stimulus timing, intensity, and duration.

Regarding tDCS to the cerebellum and spinal cord, Picelli et al. (2018) reported that the combination of cathodal tDCS and cathodal tsDCS during RAGT significantly improved walking endurance compared to the combination of anodal tDCS and cathodal tsDCS during RAGT. The cerebellum plays an important role in the coordinated movements of the limbs and posture. Cerebellar stimulation may increase inhibition of the cerebellar nuclei and decrease abnormal excitation of the cerebral cortex (Naro et al., 2017). Cathodal tsDCS has the potential to improve motor unit recruitment (Bocci et al., 2014).

We applied the PEDro scale to evaluate the risks of bias and the GRADE system to evaluate the quality of evidence. The GRADE system is currently the most widely accepted approach for grading the quality of evidence in systematic reviews and clinical practice guidelines, and for grading the strength of recommendations in clinical practice guidelines. A major strength of this study was that online and offline stimulations were assessed separately, resulting in a relatively high quality of evidence. However, given the small number of included studies, further studies are required to establish a strong evidence base.

However, this review had several limitations. First, we might not have identified all the relevant studies, as our inclusion criteria consisted of selected keywords and databases. Second, we only included studies published in English; therefore, it was unavoidable to have certain language biases and limited generalizability of these studies. Third, this meta-analysis was limited to a small number of studies to rigorously compare anodal tDCS with sham tDCS on motor-related areas of the lower limbs. Therefore, this systematic review could not draw definitive conclusions because of the limited sample size. Future studies should increase the sample size and investigate whether the combination of online tDCS and gait training could improve the walking ability. Fourth, given that the included studies differed in time since stroke onset, intervention method, stimulation site, stimulation intensity, and repetitive gait training method, drawing conclusions from the present results requires careful interpretation.

Despite these limitations, to our knowledge, this is the first report that summarizes the evidence comparing and validating online and offline stimulations for patients post-stroke. Future studies with a larger sample size and a longer follow-up period should reproduce the results to establish appropriate stimulation parameters and gait interventions to improve gait performance in patients post-stroke.

In conclusion, this systematic review and meta-analysis suggests that simultaneous application of anodal tDCS to motor-related areas of the lower limbs while repetitive gait training appears to improve walking ability more effectively. We could demonstrate important factors in tDCS intervention methods; however, we could not make definitive conclusions regarding the effects of simultaneous tDCS and gait training intervention because of the small sample size. Therefore, more high-quality studies are needed on the effects of online stimulation, including various stimulation parameters.

## Data Availability Statement

The original contributions presented in the study are included in the article/Supplementary Material, further inquiries can be directed to the corresponding author.

## Author Contributions

TM: conceptualization and writing–original draft. TM and RT: supervision. TM, TI, and TH: literature search and acquisition of data. TM, TI, TH, and RT: literature collection and writing–review and editing. TM, TI, and RT: methodology and interpretation of data. All authors contributed to the article and approved the submitted version.

## Conflict of Interest

The authors declare that the research was conducted in the absence of any commercial or financial relationships that could be construed as a potential conflict of interest.

## Publisher’s Note

All claims expressed in this article are solely those of the authors and do not necessarily represent those of their affiliated organizations, or those of the publisher, the editors and the reviewers. Any product that may be evaluated in this article, or claim that may be made by its manufacturer, is not guaranteed or endorsed by the publisher.
