# Supplementary material for: Effects of Combining Online Anodal Transcranial Direct Current Stimulation and Gait Training in Stroke Patients: A Systematic Review and Meta-Analysis
Source: Front Hum Neurosci. 2021 Dec 10;15:782305. doi: 10.3389/fnhum.2021.782305 (PMC8708562; doi:10.3389/fnhum.2021.782305)
Supplement: Supplementary file 1 [file Data_Sheet_1.docx]

Supplementary file 1. Search strategy

|  | | Search number | Query |
| --- | --- | --- | --- |
| P and I and O | | #11 | #4 AND #7 AND #10 |
| O | gait performance | #10 | #8 OR #9 |
|  |  | #9 | ((Walking[MeSH Terms]) OR Walking[Title/Abstract]) OR Ambulation[Title/Abstract] |
|  |  | #8 | ((Gait[MeSH Terms]) OR Gait[Title/Abstract]) OR Gaits[Title/Abstract] |
| I | transcranial direct current stimulation | #7 | #5 OR #6 |
|  |  | #6 | ((((((((Electric Stimulation[MeSH Terms]) OR (Electric Stimulation[Title/Abstract])) OR (Electrical Stimulation[Title/Abstract])) OR (Electrical Stimulations[Title/Abstract])) OR (Stimulation, Electrical[Title/Abstract])) OR (Stimulations, Electrical[Title/Abstract])) OR (Stimulation, Electric[Title/Abstract])) OR (Electric Stimulations[Title/Abstract])) OR (Stimulations, Electric[Title/Abstract]) |
|  |  | #5 | (((((((((((((((((((((((((Transcranial Direct Current Stimulation[MeSH Terms]) OR (Transcranial Direct Current Stimulation[Title/Abstract])) OR (tDCS[Title/Abstract])) OR (Cathodal Stimulation Transcranial Direct Current Stimulation[Title/Abstract])) OR (Cathodal Stimulation tDCS[Title/Abstract])) OR (Cathodal Stimulation tDCSs[Title/Abstract])) OR (Stimulation tDCS, Cathodal[Title/Abstract])) OR (Stimulation tDCSs, Cathodal[Title/Abstract])) OR (tDCS, Cathodal Stimulation[Title/Abstract])) OR (tDCSs, Cathodal Stimulation[Title/Abstract])) OR (Transcranial Random Noise Stimulation[Title/Abstract])) OR (Transcranial Alternating Current Stimulation[Title/Abstract])) OR (Transcranial Electrical Stimulation[Title/Abstract])) OR (Electrical Stimulation, Transcranial[Title/Abstract])) OR (Electrical Stimulations, Transcranial[Title/Abstract])) OR (Stimulation, Transcranial Electrical[Title/Abstract])) OR (Stimulations, Transcranial Electrical[Title/Abstract])) OR (Transcranial Electrical Stimulations[Title/Abstract])) OR (Anodal Stimulation Transcranial Direct Current Stimulation[Title/Abstract])) OR (Anodal Stimulation tDCS[Title/Abstract])) OR (Anodal Stimulation tDCSs[Title/Abstract])) OR (Stimulation tDCS, Anodal[Title/Abstract])) OR (Stimulation tDCSs, Anodal[Title/Abstract])) OR (tDCS, Anodal Stimulation[Title/Abstract])) OR (tDCSs, Anodal Stimulation[Title/Abstract])) OR (Repetitive Transcranial Electrical Stimulation[Title/Abstract]) |
| P | Stroke | #4 | #1 OR #2 OR #3 |
|  |  | #3 | ((Intracranial Hemorrhage[MeSH Terms]) OR Intracranial Hemorrhage[Title/Abstract]) OR Posterior Fossa Hemorrhage[Title/Abstract] |
|  |  | #2 | (((((((Brain Infarction[MeSH Terms]) OR Brain Infarction[Title/Abstract]) OR Anterior Circulation Brain Infarction[Title/Abstract]) OR Venous Infarction[Title/Abstract]) OR Brain Venous Infarction[Title/Abstract]) OR Venous Brain Infarction[Title/Abstract]) OR Anterior Cerebral Circulation Infarction[Title/Abstract]) OR Posterior Circulation Brain Infarction[Title/Abstract] |
|  |  | #1 | (((((((((((Stroke[MeSH Terms]) OR Stroke[Title/Abstract]) OR Cerebrovascular Accident[Title/Abstract]) OR CVA[Title/Abstract]) OR Cerebrovascular Apoplexy[Title/Abstract]) OR Vascular Accident[Title/Abstract]) OR Brain Vascular Accident[Title/Abstract]) OR Cerebrovascular Stroke[Title/Abstract]) OR Apoplexy[Title/Abstract]) OR Cerebral Stroke[Title/Abstract]) OR Acute Stroke[Title/Abstract]) OR Acute Cerebrovascular Accident[Title/Abstract] |
